# Managing Cow’s Milk Protein Allergy with an Extensively Hydrolyzed Formula: Results from a Prospective, Non-Interventional Study in France (EVA Study)

**DOI:** 10.3390/nu14061203

**Published:** 2022-03-12

**Authors:** Julie Lemale, Jean-Luc Decline, Catherine Dive-Pouletty, Chantal Touboul, Nadège Pichon, Christophe Dupont

**Affiliations:** 1 Paediatric Nutrition and Gastroenterology Department, Trousseau Hospital (APHP), 75 012 Paris, France; 2 Independent Researcher, 33 200 Bordeaux, France; jldecline@gmail.com; 3 Nestlé Health Science, 92 130 Issy-les-Moulineaux, France; catherine.divepouletty@fr.nestle.com (C.D.-P.); nadege.pichon@fr.nestle.com (N.P.); 4 Real World Evidence Department, Kantar Health, 75 014 Paris, France; chantal.touboul@kantarhealth.com; 5 Pediatric Department, Université Paris Descartes, Paris, France & Clinique Marcel Sembat, Ramsay Santé, 92 100 Boulogne-Billancourt, France; christophe.dupont@wanadoo.fr

**Keywords:** milk hypersensitivity, cow’s milk protein allergy, CoMiSS^®^, extensively hydrolysed formula food allergy

## Abstract

Symptoms related cow’s milk proteins allergy (CMPA) usually improve between two to four weeks following an elimination diet, firstly with extensively hydrolyzed formulas (eHF). The aim of the EVA study was to observe the evolution of CMPA-related symptoms in real life after initiation of a whey-based extensively hydrolyzed formula (w-eHF, Althéra^®^, Nestlé Health Science, Switzerland). This cross-sectional prospective non-interventional study was carried out alongside paediatricians in private practice in France between June 2019 and June 2020. Infants aged 0–3 years presenting with confirmed diagnosis or clinical symptoms suggesting CMPA were enrolled. Data were collected at enrolment (baseline visit) and three to five weeks later (follow-up visit). Symptoms were assessed using the Cow’s Milk-related Symptom Score (CoMiSS^®^). The per protocol population included 135 infants. The average number of symptoms per infant significantly decreased under the study formula (from 2.81 to 1.36, *p* < 0.001) and the proportions of infants with any CMPA related symptoms decreased. Daily crying and regurgitation showed the largest decline, respectively −44.4% and −31.85% (*p* < 0.001). These results describe the early management of symptoms suspected to be related to CMPA in routine practice that was rarely described in the literature. The number and severity of symptoms decreased most of the cases after commencing the study formula.

## 1. Introduction

Food allergy remains a major public health concern. Cow’s milk is the main food allergen in young children under three years of age [1]. The pan-European EuroPrevall birth cohort found an incidence of challenge-confirmed cow’s milk protein allergy (CMPA) of below 1% in children up to two years of age who introduced cow’s milk in the first year of their life [2]. Other studies have suggested a prevalence between 2–3% [1,2,3].

CMPA can be categorized into two different types, depending on the underlying immune mechanisms [3]. In immunoglobulin-E (IgE)-mediated CMPA symptoms appear within minutes to 1–2 h of ingestion. Common symptoms of immediate reactions include urticaria, lip swelling and facial angioedema and vomiting [4,5]. Non-IgE-mediated CMPA may be delayed by up to one week after ingestion with frequent symptoms of skin reactions and digestive manifestations occurring in 50 to 60% of patients [1,3,5].

Several recommendations and guidelines have been issued in the last decade to support the diagnostic procedure in clinical practice [1,6,7,8]. A formal diagnosis of CMPA needs to be confirmed or excluded by a sequency of allergen elimination and an oral food challenge (OFC) [9]. When CMPA is suspected in non-breastfed infants, an eHF is generally recommended as first-line formula choice, while an AAF is restricted to severe CMPA and to children who do not tolerate an eHF [10]. The diagnostic elimination diet with a close monitoring of symptoms’ evolution generally lasts between two and four weeks [11]. In France, only eHF and AAF formulas are reimbursed for CMPA by the national social insurance program. The measurement of serum specific IgE and the skin prick test to cow milk are interpreted in the context of medical history and used to support diagnosis [1].

The study formula (Althéra^®^, Nestlé Health Science, Switzerland) is an extensively hydrolysed whey-based formula (w-eHF). The safety and efficacy of the study formula have been demonstrated in several randomized controlled studies in the target population of infants with CMPA [12,13,14]. Studies of the use of eHF in routine clinical practice, outside the scope of randomized and controlled studies, are rare and there is only limited literature on the progression of CMPA symptoms after initiation of nutritional treatment with eHF.

The present observational study (Evaluation of Althéra^®^—EVA), was designed to evaluate the use of a w-eHF in the management of infants with suspected or confirmed CMPA by paediatricians in clinical practice. The aim of this study was to observe the clinical response to the w-eHF and have a better knowledge of the population of infants with CMPA managed by paediatricians in routine clinical practice. The primary objective was to analyze the progression of symptoms three to five weeks after the first prescription of the w-eHF among infants with suspected or confirmed CMPA. The secondary objectives were the description of sociodemographic characteristics, clinical profiles, and the progression of anthropometric parameters in the study population.

## 2. Materials and Methods

EVA is a cross-sectional, prospective non-interventional study analyzing the clinical profile and evolution of symptoms in infants with suspected or confirmed CMPA in routine clinical practice in France. The symptoms were assessed based either on the specific immediate (anaphylactic) symptoms seen in IgE-mediated CMPA or on the items constituting the Cow’s Milk-related Symptom Score (CoMiSS^®^), most of them mainly reflecting non-IgE-mediated CMPA (except urticaria). The CoMiSS^®^ questionnaire is an awareness tool used in primary clinical practice [15,16] to capture and score the following symptoms: duration of daily crying (score from 0 to 6), number and duration of episodes of regurgitation (score from 0 to 6), stool type according to Bristol scale (score of 0 for normal stools [type 3 and 4], score of 2 for soft stools [type 5], score of 4 for hard stools [type 1 and 2] or liquid stools [type 6] and score of 6 for watery stools [type 7]), presence and severity of atopic eczema (score from 0 to 6), urticaria (score 0 or 6) and presence and severity of respiratory symptoms (score from 0 to 3). In each of these symptoms, a score of 0 means an absent and/or normal symptom. Conversely, a higher individual score expresses a higher clinical severity, and each increase in the score means a more severe manifestation. 

Data were prospectively collected between June 2019 and June 2020.

### 2.1. Investigators

This study was carried out among pediatricians in private practice who were eligible if involved in the diagnosis and treatment of at least three infants with CMPA per month. The participating pediatrician agreed to fully meet the requirements of the study protocol.

### 2.2. Participants

Infants aged 0 to 3 years of age presenting with confirmed diagnosis or symptoms suggestive of CMPA were enrolled after an assessment by the pediatrician. The study formula was prescribed to participating infants on the day of the baseline visit. Informed consent was obtained from parents or legal guardians. The study aimed to recruit 300 infants from approximately 100 sites (three to seven patients per site).

### 2.3. Data Collection

Data were collected during two consecutive study visits: at enrolment (baseline visit) and three to five weeks later (follow-up visit), in accordance with current practice and published guidelines of Committee on Nutrition of the French Society of Paediatrics and ESPGHAN [1,10]. 

The observations focused on medical parameters and clinical history of the participants:

At baseline, pediatricians were asked to assess the most likely type of CMPA for each patient (IgE like, non-IgE like or mixed IgE/non-IgE like). The following data were collected: birth weight, birth method and gestational age, sociodemographic details (sex, age), dietary history (duration of breastfeeding, complementary feeding) and family history of allergy. Specific questions were included for subjects with IgE and mixed CMPA types (information related to consultation of an allergist and possible IgE testing). Anthropometric measurements (current height, weight, and head circumference) were assessed at baseline and follow-up visits. 

For the primary endpoint, i.e., the progression of symptoms related to CMPA between the two visits, outcomes were collected according to the classification of CMPA retained for each child. For the likely IgE-mediated CMPA, outcomes included the description of immediate allergic reactions (including urticaria, a CoMiSS^®^ item). For the likely non-IgE CMPA, outcomes were the items constituting the CoMiSS^®^ questionnaire together with rectal bleeding. For the likely mixed type CMPA, all symptoms were documented.

Regardless of CMPA type, failure to thrive as determined by the pediatrician was recorded for all patients. 

Data were collected via an electronic case report form (e-CRF) hosted on a secure web portal dedicated to the study application.

### 2.4. Statistics

Two study populations were considered in the analysis: the intent to treat (ITT) population and the per protocol (PP) population. The ITT population encompasses all infants included at baseline visit. Infants who were switched to a standard formula over the study period and infants for whom CMPA was not confirmed by the pediatrician were considered as “unlikely CMPA” and were excluded from the final analysis. The PP population was restricted to infants labeled “likely CMPA” over the study period, with valid questionnaires at baseline and at follow-up, allowing analysis of symptom progression over the study period. In the PP population, the progression of symptoms was assessed for each patient by the comparison of the number of symptoms related to CMPA and the absence/presence of symptoms before and after the formula intake. To analyze the difference in severity before and after formula intake, individual mean CoMiSS^®^ scores were compared for each individual symptom (except for urticaria) among the infants with a present/abnormal symptom (individual CoMiSS^®^ scores > 0). The analysis of quantitative variables was based on mean, standard deviation, minimum and maximum values and compared with a Student’s *t*-test or ANOVA. The Wilcoxon Mann–Whitney test was used as a non-parametric alternative to the Students *t*-test. The number and frequency of all modalities were used for the analysis of qualitative variables and were compared with a Pearson chi-square or Fisher’s exact test. For paired comparisons, paired Student’s *t*-tests for continuous variables were used. The non-parametric alternative used was the Wilcoxon test. A McNemar test was used for the comparison of paired nominal data. Mean (standard deviation) and median [min-max] values at baseline and follow-up were compared with a Wilcoxon test on dependent samples. 

Anthropometric data, including weight-for-age and height-for-age z-scores, were based on the World Health Organization (WHO) Child Growth Reference.

### 2.5. Ethical Conduct of the Study

Data of this non interventional study were available from the medical records of the patients, therefore no ethical board approval is needed. This study was conducted under the methodology MR-004 of the French data protection authority (Commission nationale de l’informatique et des libertés, CNIL). This methodology was released on 13 July 2018 and provides a legal framework for processing personal data for purposes of study, evaluation or research not involving the human person. Pediatricians have informed the parents or legal representative of the infants about the processing of personal data in compliance and within the limits of the applicable laws and regulations.

This study was sponsored by Nestlé Health Science France and was conducted by Kantar Health.

## 3. Results

### 3.1. Intent to Treat and Per Protocol Populations

The ITT population was composed of 207 infants with clinical symptoms suggesting CPMA or with a confirmed diagnosis of CMPA as recruited by 48 pediatricians. From this ITT population, 26 infants were excluded for being considered as unlikely CMPA, either according to the investigator assessment in the follow-up visit (*n* = 2) or switched back to a standard cow milk formula during the study period (24). In 46 infants, follow-up information was incomplete or unavailable.

**Figure 1:**
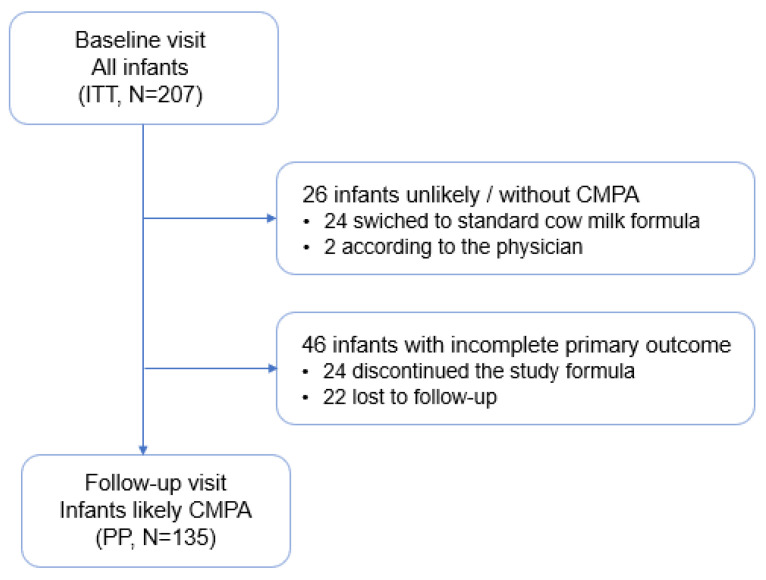
Patient flow diagram. ITT (intent to treat); PP (per protocol).

The PP population included 135 infants who had continued taking the study formula over three to five weeks and completed the baseline and follow-up visits. (Figure 1).

The patients’ characteristics of the ITT and PP population are listed in Table 1. Most of the infants were aged four months or less at inclusion (ITT 80.7%, PP 83.7%). The majority were born at term by vaginal delivery. More than half of the study population (ITT: 51.2%, PP: 50.4%) had a family history of atopy. For approximately 6% in both populations, a diagnosis of CMPA was already made (by elimination diet or a confirmation test) before enrolment. For the remaining infants, the likely diagnosis of CMPA was made according to the pediatrician’s decision and experience with CMPA, which was strongly suggested by clinical manifestations. 

According to the pediatricians, the principal signs or symptoms leading to the consideration of CMPA were mainly gastrointestinal (75% in ITT population, 81% in PP population), followed by general manifestations, such as crying, behavioral symptoms and failure to thrive (37% in ITT population, 34% in PP population) and by skin symptoms (28% in both).

### 3.2. Symptoms at Baseline

**Figure 2:**
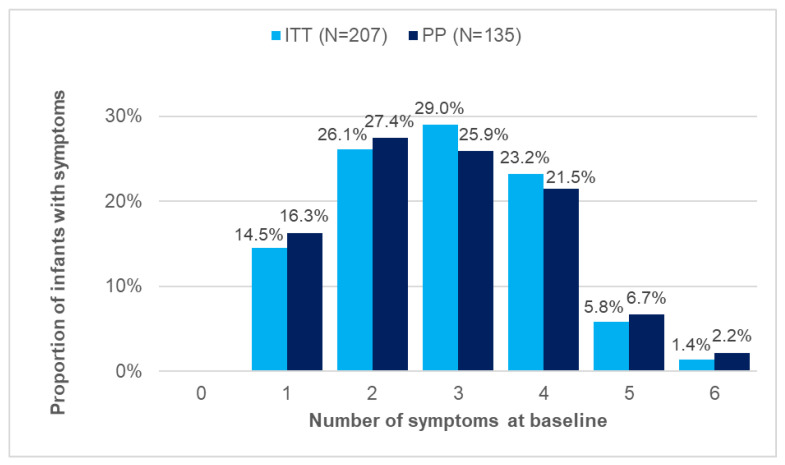
Breakdown of infants according to the number of symptoms at baseline in the ITT population (*n* = 207) and in the PP population (*n* = 135).

The distribution of number of symptoms at baseline in the ITT and PP population is shown in Figure 2. At baseline, all infants presented at least one symptom related to CMPA and the number of symptoms per infant was three on average (ITT:2.84 ± 1.19; PP: 2.81 ± 1.25). Among the PP population, most of the infants (78.3%) had between two and four symptoms (two symptoms: 26.1%, three symptoms: 29.0%, four symptoms: 23.2%).

**Figure 3:**
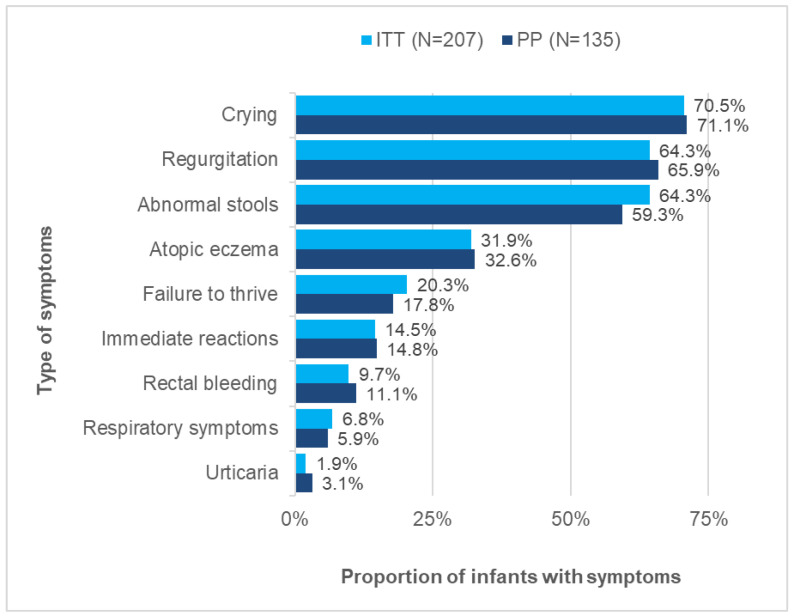
Proportion of infants presenting a symptom at baseline in the ITT population (*n* = 207) and in the PP population (*n* = 135).

Figure 3 displays the percentages of infants with CMPA symptoms. The most frequent manifestations were crying (ITT: 70.5%; PP: 71.1%), regurgitation (ITT: 64.3%; PP: 65.9%) and abnormal stools (ITT: 64.3%; PP: 59.3%). Atopic eczema was present among almost one-third of infants. Immediate (likely IgE type) reactions concerned approximately 15% of infants.

### 3.3. Progression of Symptoms from Baseline to Follow Up Visit (PP Population)

**Figure 4:**
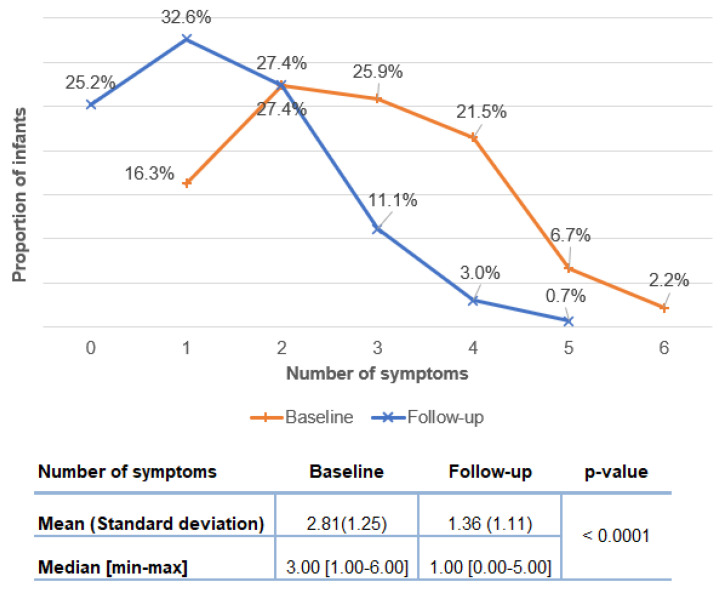
Breakdown of infants according to the number of symptoms at baseline and follow-up. The initiation of the study formula defined the “Baseline” visit and the “Follow-up” visit that was three to five weeks later. Mean (Standard deviation) and median [min-max] values at baseline and follow-up were compared with a Wilcoxon test on matched samples. *n* = 135.

The changes in symptomatology from baseline corresponding to the initiation of the study formula) to the time of follow-up visit (three to five weeks later) were analyzed based on the 135 infants of the PP population. The average number of symptoms per infant decreased significantly from 2.81 ± 1.25 to 1.36 ± 1.11 (*p* < 0.0001) and the median number of symptoms decreased significantly from 3.0 at baseline to 1.0 at follow-up (Figure 4, inserted Table). Notably, the proportion of infants with 3 and more symptoms decreased from 56.3% at base line to 14.8% at follow-up (Figure 4).

After the elimination diet with the study formula, the proportions of children affected according to the different symptoms related to CMPA decreased (Table 2). In infants likely with IgE-mediated type, immediate reactions were observed at baseline in 14.8%, and resolved for all affected infants at the follow-up visit.

In infants likely with the non-IgE-mediated CMPA type, daily crying and regurgitation showed the largest decline from baseline to follow-up (respectively −44.4% and −31.85%, *p* < 0.001). More than half of the infants (59.3%) had abnormal stools at baseline compared to 43% at follow-up. A significant drop was observed in rectal bleeding (−9.63%, *p* < 0.01) and atopic eczema (−8.89%, *p* < 0.05), as well as the disappearance of all immediate like reactions and urticaria. Overall, most infants had between 2 and 4 symptoms at baseline, while at follow-up, a complete resolution of CMPA-related symptoms was recorded in one out of four infants.

As described in the Methods section, the CoMiSS^®^ tool, intended for infants with CMPA, largely scoring symptoms of the non-IgE-mediated type, was used in 124 patients from the PP population. Among these infants, a decrease in the severity of at least one symptom was observed between baseline and follow-up visits in 90.3% of patients. When we assessed the progression of individual CoMiSS^®^ scores per symptom among infants presenting each symptom at baseline and at follow-up, a significant decrease was observed for several symptoms, notably for crying (3.27 ± 1.54 to 1,69 ± 1.54, *p* < 0.001) and atopic eczema (2.84 ± 1.41 to 1.94 ± 1.0, *p* < 0.001) (Table 2).

**Figure 5:**
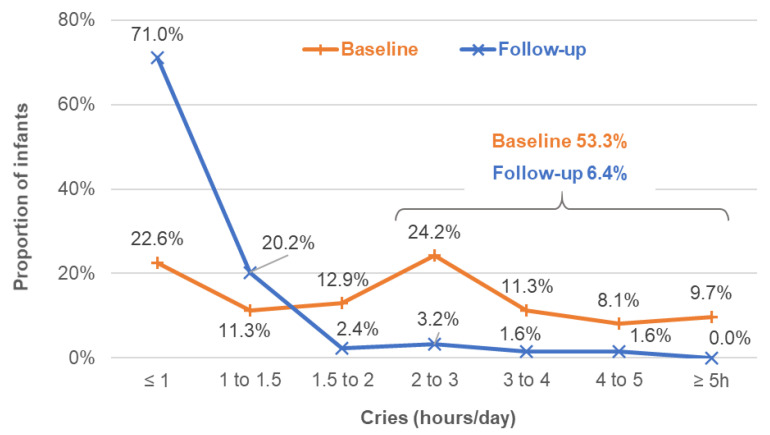
Breakdown of infants according to the frequency of crying per day at baseline and follow-up (*n* = 124 infants non-IgE or mixed like type of CMPA).

At baseline, more than half of infants (53.3%) cried more than 2 h per day, whereas only 6.4% were at follow-up. Almost 10% of infants experienced the severest level of daily crying (5 h and more) at baseline, whereas at follow-up, no more infants cried for more than 5 h per day (Figure 5). The proportion of infants crying within non--pathologic ranges (1.5 h or less) increased from 33.9% at baseline to 91.2% at follow-up.

Growth parameters were evaluated as z-scores according to the WHO Child Growth Standards at inclusion; the mean weight-for-age and height-for-age z-scores were negative and varied between −0.2 and −0.6 in the PP population, which is in the normal range. These scores remained stable and within normal values over the period of the study formula intake for the 135 infants. Among girls of the PP population, the weight-for-age z-score (mean, [95% CI]; standard deviation) varied from (−0.5 [−0.8; −0.2]; 1.18) at baseline to (−0.3 [−0.6; 0.0]; 1.15) at follow-up; height-for-age z-score (mean, [95% CI]; standard deviation) varied from (−0.2 [−0.5; 0.1]; 1.14) at baseline to (−0.2 [−0.5; 0.1]; 1.19) at follow-up. Similarly, for boys of the PP population, the weight-for-age z-score varied from (−0.6 [−0.8; −0.3]; 1.10) at baseline to (−0.6 [−0.9; −0.3]; 1.15) at follow-up; height-for-age z-score was (−0.5 [−0.7; −0.3]; 1.00) at baseline to (−0.4 [−0.6; −0.1]; 1.09) at follow-up.

At the end the follow-up visit, 92% (*n* = 124) of the population was continuing with the study formula.

## 4. Discussion

This cross-sectional prospective non-interventional study analyzed a population of infants with suspected or confirmed CMPA among which we observed a favorable outcome under w-eHF, with a decrease in the number, frequency and severity of symptoms between the initial and the follow-up consultation. This study is based on the individual items of the CoMiSS^®^ questionnaire, which is designed to be an awareness tool to capture and score CMPA-related symptoms. Unlike in a recent work [17], this study does not aim for a validation of the CoMiSS^®^. According to the protocol of the present study, CMPA-related symptoms were monitored and analyzed using individual items of the CoMiSS^®^ questionnaire between the initiation and the follow-up visits in children who were likely to have CMPA. This symptom-based approach, like in the work of Vandenplas et al. [18], reflects the real-life management of CMPA. This study provides new data on a symptom-based approach of the CoMiSS^®^ tool and on its evolution in infants initiated with an eHF formula in the routine practice of primary care paediatricians.

The inclusion of infants in the study was mainly based on symptoms suggestive of CPMA according to the pediatrician’s assessment. The diagnosis of CMPA was confirmed by a skin test or an elimination diet in a minority of infants of the study population (less than 6%). CMPA was suspected among 94% of enrolled infants based on the presence of one or more suggestive signs and symptoms. The prevalence of an OFC proven food allergy as reported in the literature ranged from 1 to 10% in infants and children under five years of age [19]. Although the challenge test is considered the gold standard in the diagnosis of CMPA, it is often not performed due to the parents’ reluctance after the significant decrease of symptoms subsequent to the elimination diet and the fear of the reappearance of symptoms [11]. Moreover, the clinical reaction occurring during or after a challenge is often difficult to interpret, especially in non-IgE-mediated CMPA [20]. In cases with severe CMPA, the challenge should be performed in a hospital setting and may even be contraindicated in case of a history of life-threatening reactions [11]. The lack of optimal diagnostic tests and reliable biomarkers makes the diagnosis of CMPA particularly challenging, in addition to the broad spectrum of signs and symptoms affecting different organ systems and the possible overlap with other conditions, infections or gastrointestinal disorders [20,21]. Unfortunately, no signs or symptoms are pathognomonic to CMPA [6]. According to the ESPGHAN recommendations, the initial step in the diagnostic work-up for CMPA should rely on the clinician’s assessment accompanied by a risk factors investigation, such as family history of atopic disease [1], and whether the infant suffers from concurrent conditions [6].

The eHF-type study formula was introduced according to international guidelines and routine practices in France where eHF is reimbursed by social insurance. Partially hydrolyzed formulas are considered unsuitable for the treatment of cow’s milk protein allergy by all international guidelines. Soy formulas are not recommended for children under six months and are not commercialized in France [1,11]. Rice formulas are nutritionally equivalent to eHF-type formulas but not reimbursed in France for CMPA.

In our study population, perinatal characteristics depicted were consistent with most recent French national statistics: in 2016, 20% of newborns were delivered via Cesarean section [22], and 56% of infants breastfed for at least a week [23]. These findings are also similar to CMPA clinical research studies, in which the levels of C sections do not usually exceed 15% [18,21]. Furthermore, our results suggest a slight yet present gender ratio imbalance favoring males, also seen in similar clinical trials [20,21]. Moreover, clinical characteristics at baseline of our study population are consistent with the publication of Salvatore et al. [20]., where data were collected in a similar prospective observational design, although hospital based. In both studies, the most frequent symptoms followed the same descending order, i.e., crying, regurgitations, abnormal stools and skin symptoms [20].

Our results present some variation compared to other published data, probably because of the scarcity of epidemiologic data on non-IgE-mediated CMPA. In a recent paper, CMPA was reported to be IgE-mediated in 50% to 60% of cases [11,24], and cutaneous and respiratory symptoms seem to appear in 50–60% and 20–30% of cases, respectively [6]. The higher proportion of likely non-IgE types of CMPA and the lower proportion of respiratory and cutaneous manifestations, especially urticaria, observed in our study population probably relates to the fact that non-IgE-mediated CMPA actually constitutes the main presentation of CMPA in pediatric practice and is handled mainly by the pediatrician. In contrast, IgE type reactions, which can be life threatening [6], are usually managed by an allergist. Similarly, patients with skin manifestations frequently refer to dermatologists in a specialized secondary care setting and are therefore seen less by pediatricians. Moreover, ESPGHAN stated that it is usually not possible to distinguish the IgE and non-IgE types of CMPA in clinical practice solely on physical examination [1], which suggests a possible misclassification of CMPA types of allergy in some patients. This is why there may be a bias in the epidemiological data, as pointed out by Flom JD et al. [24].

Our study provides recent insights on the progression of the CMPA symptoms three to five weeks after a first prescription of the w-eHF Althéra^®^, according to the evolution of CoMiSS^®^ components as well as other symptoms, such as rectal bleeding, failure to thrive and immediate IgE-like type reaction. The CoMiSS^®^ is a six item symptom-based score originally designed to assess the evolution of CMPA symptoms during dietary intervention [21,25]. The present results suggest an improvement of symptomatology in our PP population before and after Althéra^®^ intake, with both a significant decrease in the total number of symptoms per infant and in the severity of all symptoms (except respiratory symptoms) related to CMPA in the clinical picture. The severity of several symptoms decreased significantly as well, especially the duration of daily crying, the number of daily episodes of regurgitations, as well as atopic eczema manifestations. The finding relating to daily crying shows simultaneously the largest decrease of the proportion of infants affected within the study population and of the duration of daily average crying.

According to ESPGHAN GI Committee Practical Guidelines, the duration of an elimination diet depends on clinical manifestations and should be kept as short as possible, yet long enough to fully appreciate if symptoms resolve or not or stabilize [1]. In children with immediate types of reactions, three to five days of a cow milk free diet are sufficient to judge the response. However, a longer duration is necessary for patients with delayed clinical reactions: one to two weeks for eczema and rectal bleeding, and two to four weeks for gastro-intestinal manifestations. In case of persistent symptoms with no clinical improvement, infants should be switched to AAF before the exclusion of CMPA [1]. In our study population, we observed a resolution of all immediate reactions.

### Limits of the Study

The primary endpoint of this trial was to analyze the progression of symptoms three to five weeks after the first prescription of the w-eHF among infants with suspected or confirmed CMPA. The number of children enrolled allowed us to reach clinical significance for this primary endpoint. However, the relevance of these data rely on the perception of CMPA and the coding of the clinical symptoms that largely depend on the geographical and/or medical setting. Therefore, caution is deemed necessary when drawing conclusions for children in a different geographical and/or medical environment.

The observation lasted for three to five weeks, which may be shorter than the natural evolution of some symptoms associated with progressive cow’s milk proteins tolerance acquisition. Regarding persistent delayed manifestations in our study population, either the duration of the elimination diet was not long enough to record an improvement, or the symptoms were particularly resistant to w-eHF and needed to be treated with AAF, or finally CMPA should have been ruled out by the pediatrician and a differential diagnosis considered.

Although anthropometric data were overall normal at baseline and remained stable at follow-up visit, several clinical studies suggest a minimal period observation of one month up to six months to achieve a significant increase in growth indicators [26]. The study period was perhaps insufficient to observe a complete normalization of infants who were stunted or underweight according to the growth WHO standards. However, significant improvement of growth was observed among infants with reported failure to thrive at baseline.

In the present study, most of the infants were under four months of age at inclusion. Even though cow milk allergy usually does not develop in infant above one year old [11], our study population seems to be very young and can possibly represent only a subset of infants suffering from CMPA. The study set-up did not include hospitals and secondary care. Thus, the study population probably did not include all CPMA profiles, especially the ones with specific or severe manifestations.

## 5. Conclusions

This study provided recent evidence on the real-world management of CMPA in primary care in France as well as on the real-life progression of CMPA symptoms three to five weeks after a first prescription of the study formula. These results describe the early management of symptoms suspected to be related to CMPA in routine practice that was rarely described in the literature. A favorable evolution of CMPA-related symptoms was observed in infants receiving w-eHF, with a decrease in the number, frequency and severity of symptoms. At baseline, most infants had between two and four symptoms, while at follow-up, one in four infants showed a complete clearance of CMPA related symptoms and a decrease in the intensity of symptoms is observed in 90% of infants. All of the most common symptoms improved; the largest improvement was seen in the duration of daily crying and regurgitations. A significant decrease was also recorded in atopic eczema, rectal bleeding, and abnormal stools. Urticaria and immediate reactions resolved in all infants.

## Figures and Tables

**Table 1:** Characteristics of infants at baseline in the ITT and PP populations.

Item	ITT Population (N = 207)	PP Population (N = 135)
**Demographics**		
Male % (n)	56.0% (116)	56.3% (76)
Female % (n)	44.0% (91)	43.7% (59)
Age at enrolment (months)		
- Median [min-max]	3.0 [0.5–23]	3.0 [0.5–17]
- Mean (SD)	3.4 (2.8)	3.1 (2.3)
**Birth history**		
Gestational age		
- At term (37–41 weeks) % (n)	96.6% (200)	96.3% (130)
- Preterm % (n)	3.4% (7)	3.7% (5)
Delivery mode		
- Vaginal% (n)	81.2% (168)	79.3% (107)
- Cesarean section % (n)	17.9% (37)	20.0% (27)
- NA % (n)	1.0% (2)	0.7% (1)
Birth weight (kg)		
- Median [min-max]	3.2 [1.59–4.86]	3.1 [1.59–4.86]
- Mean (SD)	3.2 (0.48)	3.2 (0.48)
**Anthropometrics parameters at baseline**		
Weight (kg)		
- Median [min-max]	5.4 [2.92–12.01]	5.4 [2.92–11.56]
- Mean (SD)	5.7 (1.6)	5.6 (1.5)
Weight for age Z-scores		
- Mean [95% CI] (SD) boys	−0.5 [−0.7; −0.3] (1.00)	−0.6 [−0.8; −0.3] (1.10)
- Mean [95% CI] (SD) girls	−0.6 [−0.8; −0.4] (1.08)	−0.5 [−0.8; −0.2] (1.18)
Height (cm)		
- Median [min-max]	59.0 [49.0–87.0]	59.0 [49.0–81.0]
- Mean (SD)	59.7 (6.2)	59.4 (5.7)
Height for age Z-scores		
- Mean [95% CI] (SD) boys	−0.5 [−0.7; −0.3] (1.04)	−0.5 [−0.7; −0.3] (1.00)
- Mean [95% CI] (SD) girls	−0.3 [−0.6; −0.1] (1.18)	−0.2 [−0.5; 0.1] (1.14)
Head circumference (cm)		
- Median [min-max]	40.0 [35.0–50.0]	40.0 [35.0–49.5]
- Mean (SD)	40.3 (2.9)	40.2 (2.8)
**Dietary history**		
Breast feeding		
- Exclusive % (n)	27.1% (56)	31.1% (42)
- Mixed % (n)	26.1% (54)	26.7% (36)
Diversification of infants of at least 4 months of age % (n)	63.1% (41/65)	62.5% (25/40)
**History of CMPA**		
Diagnosis of CMPA		
- Confirmed (with a skin test or after OFC) % (n)	5.8% (12)	5.9% (8)
- Suspected by the clinical symptoms % (n)	94.2% (195)	94.1% (127)
Major sign/symptom leading to CMPA diagnosis		
- Digestive * % (n)	74.9% (155)	80.7% (109)
- General * % (n)	36.7% (76)	34.1% (46)
- Skin condition * % (n)	28.0% (58)	28.1% (38)
CMPA type		
- IgE like % (n)	9.2% (19)	8.1% (11)
- Non-IgE like % (n)	85.5% (177)	85.2% (115)
- Mixed like % (n)	5.3% (11)	6.7% (9)

* Digestive symptoms include abnormal stools, digestive disorders, gastroesophageal reflux, regurgitations, nausea/vomiting; general symptoms include crying, behavioral disorders, failure to thrive; skin conditions include eczema, urticaria. ITT, intent to treat; PP, per protocol.

**Table 2:** Frequency of symptoms and CoMiSS^®^ score per symptom at baseline and follow-up.

Frequency (Number) and Individual CoMiSS Scores Per Symptoms	Baseline PP	Follow-Up PP	From Baseline to Follow-Up	*p*-Value
**Crying**				
% (n) CoMiSS^®^ score (SD)	71.1% (96) 3.27 (1.54)	26.7% (36) 1.69 (1.20)	−44.44% −1.58	<0.001 <0.001
**Regurgitations**				
% (n) CoMiSS^®^ score (SD)	65.9% (89) 2.13 (1.19)	34.1% (46) 1.35 (0.87)	−31.85% −0.79	<0.001 <0.001
**Abnormal Stools**				
% (n) CoMiSS^®^ score (SD)	59.3% (80) 3.40 (1.36)	43.0% (58) 2.69 (1.26)	−16.30% −0.71	<0.05 <0.001
**Atopic eczema**				
% (n) CoMiSS^®^ score (SD)	32.6% (44) 2.84 (1.41)	23.7% (32) 1.94 (1.0)	−8.89% −0.86	<0.05 <0.01
**Urticaria**				
% (n) CoMiSS^®^ score (SD)	3.0% (4) 6.00 (0)	0.0% (0) 0	−2.96% −6.00	- -
**Respiratory symptoms**				
% (n) CoMiSS^®^ score (SD)	5.9% (8) 1.25 (0.43)	3.7% (5) 1.60 (0.80)	−2.22% +0.35	- -
**Immediate reaction,** % (n)	14.8% (22)	0.0% (0)	−14.81%	<0.001
**Failure to thrive,** % (n)	17.8% (24)	3.7% (5)	−14.07%	<0.001
**Rectal bleeding,** % (n)	11.1% (15)	1.5% (2)	−9.63%	<0.01
**Absence of symptoms,** % (n)	0.0% (0)	25.2% (35)	+25.2%	<0.001

Frequency of symptoms at baseline and follow-up were compared with a McNemar test. Values of the CoMiSS^®^ score per symptom at baseline and follow-up were compared with a Wilcoxon Mann Whitney. *n* = 135.

## Data Availability

The data presented in this study are available on reasonable request from the corresponding author. The data are not publicly available due to the data containing information that could compromise participant privacy.
